# Microbial community function and biomarker discovery in the human microbiome

**DOI:** 10.1186/gb-2011-12-s1-p47

**Published:** 2011-09-19

**Authors:** Nicola Segata, Sahar Abubucker, Johannes Goll, Alyxandria M Schubert, Jacques Izard, Brandi L Cantarel, Beltran Rodriguez-Mueller, Levi Waldron, Jeremy Zucker, Mathangi Thiagarajan, Bernard Henrissat, Owen White, Scott T Kelley, Barbara Methé, Patrick D Schloss, Wendy S Garrett, Dirk Gevers, Makedonka Mitreva, Curtis Huttenhower

**Affiliations:** 1 Harvard School of Public Health, Biostatistics, 655 Huntington Avenue, Boston, 02115, USA

## 

Microbial communities carry out the majority of the biochemical activity on the planet, and they play integral roles in processes such as metabolism and immune homeostasis in the human microbiome. Whole genome shotgun sequencing of such communities’ metagenomes is becoming an increasingly feasible complement to obtaining organismal information from taxonomic markers. However, the resultant dataset typically comprises short reads from hundreds of different organisms, making it challenging to assemble and functionally annotate these sequences in the standard manner for single-organism genomes.

We describe an alternative to this approach to infer the functional and metabolic potential of a microbial community metagenome by determining whether gene families and pathways are present or absent, as well as their relative abundances, directly from short sequence reads. We validated this methodology using synthetic metagenomes, recovering the presence and abundance of large pathways and of small functional modules with high accuracy. We subsequently applied this approach to the microbial communities of 649 metagenomes drawn from 7 primary body sites on 102 individuals as part of the Human Microbiome Project (HMP), demonstrating the scalability of our methodology and the critical importance of microbial metabolism in the human microbiota. This provided a framework in which to define functional diversity in comparison to organismal ecology, including an example of microbial metabolism linked to specific organisms and to host phenotype (vaginal pH) in the posterior fornix. We provide profiles of 168 functional modules and 196 metabolic pathways that were determined to be specific to one or more niches within the human microbiome, including details of glycosaminoglycan degradation in the gut.

Understanding how and why these biomolecular activities differ among environmental conditions or disease phenotypes is, more broadly, one of the central questions addressed by high-throughput biology. We have thus developed the linear discriminant analysis (LDA) effect size algorithm (LEfSe) to discover and explain microbial and functional biomarkers in the human microbiota and other microbiomes. We demonstrate this method to be effective for mining human microbiomes for metagenomic biomarkers associated with mucosal tissues and with different levels of oxygen availability. Similarly, when applied to 16S rRNA gene data from a murine ulcerative colitis gut community, LEfSe confirms the key role played by *Bifidobacterium* in this disease and suggests the involvement of additional clades, including the Clostridia and *Metascardovia*. A quantitative validation of LEfSe highlights a lower false positive rate, consistent ranking of biomarker relevance, and concise representations of taxonomic and functional shifts in microbial communities associated with environmental conditions or disease phenotypes.

Implementations of both methodologies are available at the Huttenhower laboratory’s website [1,2]. Together, they provide a way to accurately and efficiently characterize microbial metabolic pathways and functional modules directly from high-throughput sequencing reads and, subsequently, to identify organisms, genes or pathways that consistently explain the differences between two or more microbial communities. This has allowed the determination of community roles in the HMP cohort, as well as their niche and population specificity, which we anticipate will be applicable to future metagenomic studies.
